# Composition, Sources, and Health Risks of Polycyclic Aromatic Hydrocarbons in Commonly Consumed Fish and Crayfish from Caohai Lake, Southwest China

**DOI:** 10.3390/toxics13121086

**Published:** 2025-12-17

**Authors:** Yupei Hao, Tianyao Yang, Xueqin Wei, Xu Zhang, Xiongyi Miao, Gaohai Xu, Sheping Yang, Xiaohua Zhou, Huifang Zhao, Wei Bao

**Affiliations:** 1 Department of Modern Engineering, Anshun Technical College, Anshun 561000, China; yphao66@126.com; 2 Yunnan Provincial Bureau of Geology and Mineral Exploration and Development Center Laboratory & Key Laboratory of Sanjiang Metallogeny & Resources Exploration and Utilization Ministry of Natural Resources, Kunming 650051, China; yangty515@163.com (T.Y.); weixueqin@gznu.edu.cn (X.W.); yangsheping@126.com (S.Y.); zhouxiaohua@126.com (X.Z.); 3 School of Geography and Environmental Science & School of Karst Science, Guizhou Normal University, Guiyang 550001, China; zhaohuifang@gznu.edu.cn; 4 Guizhou Caohai Wetland Ecosystem Observation and Research Station, Guizhou Academy of Forestry, Guiyang 550005, China; zhangxu@126.com; 5 Nanjiang Hydrogeological & Engineering Geology Brigade, Chongqing 401121, China; 6 Yunnan Key Laboratory of Sanjiang Metallogeny and Resources Exploration and Utilization, Kunming 650051, China; 7 College of Environmental Science and Engineering, Ocean University of China, Qingdao 266100, China; baowei@stu.ouc.edu.cn

**Keywords:** polycyclic aromatic hydrocarbons, aquatic products, source identification, health risk assessment, plateau lake

## Abstract

This study investigated the occurrence, sources, and health risks of 16 polycyclic aromatic hydrocarbons (PAHs) in commonly consumed fish and crayfish from the Caohai Lake, a typical plateau lake in southwest China. Four dominant species (crucian carp, common carp, yellow catfish, and crayfish) were collected and analyzed. The results showed a generally low level of PAH contamination (mean: 26.7 μg/kg wet weight), with bioaccumulation tendency decreasing as the number of PAH rings increased. Crayfish exhibited the highest total concentration of PAHs, whereas yellow catfish accumulated the most carcinogenic PAHs. Positive matrix factorization (PMF) model identified four primary sources—petroleum leakage, coal combustion, traffic emissions, and biomass burning—with petroleum-derived PAHs being the most significant contributor. The assessment of health risk indicated that while the average hazard index (HI) was below 1, approximately 10% of the samples posed a potential non-carcinogenic risk, particularly from crayfish and yellow catfish. The incremental lifetime cancer risk (ILCR) for DahA, BaP, BaA, and BbF all exceeded the negligible risk level of 10^−6^ but remained below 10^−4^. Notably, the mean total ILCR (TILCR) approached 10^−4^, with yellow catfish presenting the highest carcinogenic risk, highlighting concerns of the carcinogenic risk of PAHs. Source-oriented risk assessment revealed that petroleum leakage was the dominant contributor to non-carcinogenic risk (>55%), while traffic emissions contributed most to carcinogenic risk (>57%). To mitigate carcinogenic risk, implementing stormwater diversion systems along the circular lakeside roads is recommended to reduce the input of traffic-derived PAHs.

## 1. Introduction

Polycyclic aromatic hydrocarbons (PAHs), a group of persistent organic pollutants characterized by two or more fused aromatic rings, have raised global concern due to their persistence, long-range transport potential, and detrimental health effects, including carcinogenicity, mutagenicity, and teratogenicity. Consequently, 16 PAHs have been designated as priority pollutants by the United States Environmental Protection Agency (USEPA) [1,2,3]. These compounds primarily originate from the incomplete combustion of fossil fuels and biomass, as well as petroleum leakage, and are subsequently released into the environment through various pathways [3,4]. With the continuous increase in global energy consumption, the emission of PAHs and their associated health risks remain a significant public health issue [5,6].

Aquatic products, such as fish and shrimp, represent a crucial source of high-quality protein, unsaturated fatty acids, and trace elements for humans, with superior digestibility compared to livestock meat [7,8]. While their consumption offers recognized health benefits, it also poses potential risks due to the bioaccumulation of environmental contaminants [9,10]. Historically, research on contaminant accumulation in aquatic products has predominantly focused on heavy metals [11,12]. In contrast, the status of PAHs, another prevalent class of environmental contaminants [6], has received considerably less attention. Given their pronounced lipophilicity, PAHs possess a high potential for bioaccumulation in aquatic organisms [13]. Therefore, investigating the levels, sources, and health risks of PAHs in aquatic products is essential for a comprehensive dietary risk assessment.

The Caohai Lake, a typical plateau lake in southwestern China and a vital wintering and stopover site for migratory waterbirds, is facing increasing environmental pressures [14]. Its proximity to Weining County has made it a sink for PAHs derived from historical energy-intensive industries (e.g., zinc smelting that heavily rely on coal use), domestic sewage, and agricultural runoff [9,10]. Previous studies have confirmed the presence of PAHs in environmental matrices such as soil, water, and sediments within the watershed of Caohai Lake [15,16]. However, these investigations have predominantly concentrated on abiotic media, leaving a significant knowledge gap regarding the bioaccumulation of PAHs in aquatic organisms, which is a critical link for assessing ecological and human health risks. The toxicity of PAHs is closely related to their biotransformation [17], making it imperative to study their bioaccumulation in biota to fully understand the long-term ecological effects of PAH pollution in the watershed.

To address this gap, this study selected four dominant aquatic species in Caohai Lake—yellow catfish (*Pelteobagrus fulvidraco*), crucian carp (*Carassius auratus*), common carp (*Cyprinus carpio*), and crayfish (*Procambarus clarkii*)—as study objects. The objectives were to (1) determine the concentration and composition profiles of 16 USEPA priority PAHs in these species; (2) identify and apportion the potential sources of the accumulated PAHs; and (3) quantitatively evaluate the associated carcinogenic and non-carcinogenic health risks. The findings aim to provide a scientific basis for targeted risk management and pollution control strategies in the Caohai watershed.

## 2. Material and Methods

### 2.1. Study Sites

**Figure 1:**
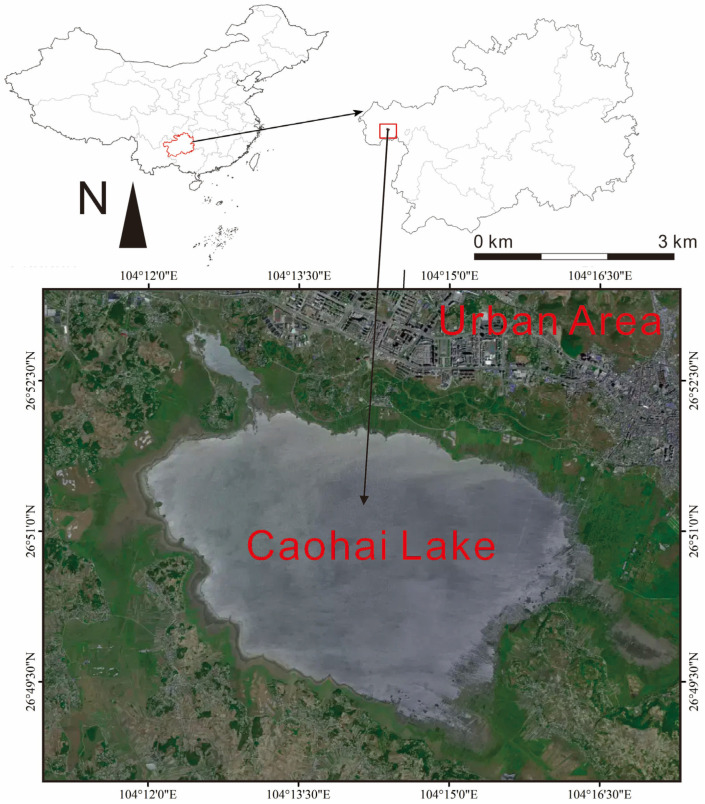
The location of Caohai Lake in Guizhou Province and China.

Caohai Lake is located in Weining County, Bijie City, in the northwest of Guizhou Province (Figure 1). Caohai Lake is an important plateau lake in southwestern China with the altitude exceeding 2200 m. The Nature Reserve of Caohai Lake was upgraded from provincial-level to national-level to accelerate the ecological protection of Caohai Lake in 1998, which covers a total area of approximately 120 km^2^ [18]. The northeast part of Caohai Lake reserve is adjacent to the old urban area of Weining County, which possesses prosperous municipal and industrial operations, while the remainder of the Caohai Lake reserve is mainly within the areas of forests, shrubs and arable lands [19]. Due to seasonal precipitation, the water surface area fluctuates between 19.8 km^2^ during the dry season and 26.0 km^2^ in the wet season, with a maximum water depth of 5.3 m and an average depth of 1.6 m [20,21]. The region experiences a mean annual temperature of 10.5 °C and receives an average annual rainfall of 951 mm. Historical data indicate that the Caohai Lake is dominated by small fish species characterized by rapid growth rates [22,23]. Crucian carp (*Carassius auratus*), common carp (*Cyprinus carpio*), and yellow catfish (*Pelteobagrus fulvidraco*) are the dominant fish species in the area. The crayfish (*Procambarus clarkii*), introduced into Caohai Lake in 2012 for aquacultural purposes, has since undergone rapid population expansion and now proliferates throughout the lake, becoming the most ecologically threatening invasive species in the ecosystem of Caohai Lake [22]. Crucian carp, common carp, yellow catfish and crayfish are the commonly consumed aquatic species in Caohai Lake.

### 2.2. The Collection, Preparation and Analysis of the Samples

This study focused on four dominant aquatic species in the watershed of Caohai Lake, crucian carp (*Carassius auratus*), common carp (*Cyprinus carpio*), yellow catfish (*Pelteobagrus fulvidraco*), and crayfish (*Procambarus clarkii*), and crayfish (*Procambarus clarkii*), all of which were selected for their high economic value and widespread distribution. Sampling was conducted in July 2025 using gillnets and ground cages. Upon collection, the specimens were immediately labeled, placed in sealed plastic bags, and transported under frozen conditions to the laboratory. In the laboratory, species identification was confirmed, and body weight and length were measured. The detailed biological parameters are summarized in Table 1.

Following species identification and morphometric measurements, the fish and crayfish specimens were dissected. After removing the scales, a 50–100 g sample of dorsal muscle tissue was excised from each individual fish. For smaller specimens, muscle tissues from three fish of comparable size and from the same sampling location were pooled to meet the minimum sample weight requirement. The edible parts of crayfish (tail) were collected after removing their heads and shells. The prepared muscle samples were rinsed with deionized water, weighed, and then lyophilized at −80 °C for 72 h until a constant weight was achieved. The freeze-dried tissues were subsequently ground into a homogeneous powder, sealed in plastic bags, and stored frozen until chemical analysis.

For PAH analysis, a 10 g aliquot of the powdered fish sample was spiked with 200 ng of a deuterated PAH surrogate standard solution and thoroughly mixed with an equal amount of anhydrous sodium sulfate. The homogenate was then subjected to ultrasonically assisted extraction with 30 mL of an acetone/dichloromethane/n-hexane mixture (1:1:1, *v*/*v*/*v*) at 30 °C for 30 min. After filtration, the extraction was repeated twice with fresh solvent. The combined extracts were concentrated to approximately 2 mL using a rotary evaporator (30 °C, 250 mbar) and subsequently purified on a chromatography column packed with 2.0 g of silica gel and 2.0 g of alumina. The PAHs were eluted with 25 mL of n-hexane/dichloromethane (9:1, *v*/*v*), and the eluate was gently concentrated under a pure nitrogen stream to a final volume of 2 mL for instrumental analysis.

The cleaned extracts were analyzed for PAHs using an Agilent 6890 gas chromatograph (Santa Clara, CA, USA) coupled with a 5975 mass spectrometer (GC-MS). Separation was achieved on a DB-5 capillary column (30 m × 0.25 mm i.d. × 0.25 μm film thickness; J&W Scientific, Folsom, VA, USA). The GC oven temperature program was set as follows: initial hold at 50 °C for 4 min, increased to 200 °C at a rate of 10 °C min^−1^, then to 240 °C at 5 °C min^−1^, and further to 290 °C at 3 °C min^−1^, and a final hold at 290 °C for 3 min. A 1.0 μL aliquot of the sample was injected in pulsed splitless mode. The injector and ion source temperatures were maintained at 280 °C and 240 °C, respectively. Ionization was conducted via electron impact (EI) at 70 eV, and data were acquired in the selective ion monitoring (SIM) mode. PAHs were identified by comparing the relative retention times and the ratios of target to qualification ions against those of authentic external standards.

### 2.3. Quality Assurance and Quality Control

Field and method blanks were used to validate the validation of the extraction procedure and determine the recoveries of the deuterated and undeuterated PAHs. The recoveries for the deuterated PAHs and undeuterated standard PAHs respectively ranged from 89 to 102% and 82 to 97.8%. No PAHs were detected in the method blanks (*n* = 5). Triplicate testing was carried out in each sample to maintain the precisions of relative standard deviation (RSD) within the range of 0.5 to 3%. An external calibration method was used to quantify the concentrations of PAH in the samples. Of the 16 USEPA priority PAHs, the method detection limit (LOD) and quantification limit (LOQ) for Naphthalene were 0.11 µg/L and 0.33 µg/L, respectively. For Acenaphthylene, the LOD and LOQ were 0.26 µg/L and 0.78 µg/L; for Acenaphthene, 0.31 µg/L and 0.93 µg/L; for Fluorene, 0.42 µg/L and 1.26 µg/L; for Phenanthrene, 0.28 µg/L and 0.84 µg/L; and for Anthracene, 0.15 µg/L and 0.45 µg/L. The LOD and LOQ for Fluoranthene were 0.30 µg/L and 0.90 µg/L; for Pyrene, 0.18 µg/L and 0.54 µg/L; for Benzo[a]anthracene, 0.21 µg/L and 0.63 µg/L; and for Chrysene, 0.15 µg/L and 0.45 µg/L. For Benzo[b]fluoranthene, the LOD and LOQ were 0.16 µg/L and 0.48 µg/L; for Benzo[k]fluoranthene, 0.11 µg/L and 0.33 µg/L; for Benzo[a]pyrene, 0.10 µg/L and 0.30 µg/L; and for Dibenzo[a,h]anthracene, 0.31 µg/L and 0.93 µg/L. Finally, for Benzo[g,h,i]perylene, the LOD and LOQ were 0.50 µg/L and 1.50 µg/L, and for Indeno [1,2,3-c,d]pyrene, they were 0.63 µg/L and 1.89 µg/L.

### 2.4. The Health Risk Assessment of PAHs

The health risks associated with PAHs, encompassing both carcinogenic and non-carcinogenic effects, were assessed following the framework recommended by the US EPA [24]. For carcinogenic risk, the Incremental Lifetime Cancer Risk (ILCR) was adopted, which quantifies the probability of an individual developing cancer over a lifetime due to exposure to environmental pollutants [25,26]. The total incremental lifetime cancer risk (TILCR) was calculated as the sum of the ILCR values for all individual PAHs. Prior to the ILCR calculation, the benzo[a]pyrene equivalent concentration (BaP_eq_) was determined for each sample using the toxicity equivalence factors (TEFs) of the constituent PAHs (Table 2), according to the following Formulas ((1)–(3)):

(1) $${BaP}_{eq}=C_{i}\times {TEF}_{i}$$

(2) $$ILCR=\frac{{BaP}_{eq}\times CSF\times IR\times EF\times ED}{BW\times AT}$$

(3) $$TILCR=\sum_{i=1}^{16}ILCR$$

In the equations, C_i_ represents the concentration of PAH congener i in the organisms (ng·g^−1^, wet weight), and TEF_i_ is its corresponding toxic equivalency factor. The TEF values applied in this study (Table 3) are based on the carcinogenic potency of individual PAHs. The carcinogenic slope factor (*CSF*) was set at 7.3 mg·kg^−1^·day^−1^. Population-specific exposure parameters, including body weight (*BW*), exposure duration (*ED*), and intake rate (*IR*), were derived from the Exposure Factors Handbook of Chinese Population [29]. Other fixed parameters were defined as follows: exposure frequency (*EF*) = 365 days·year^−1^ and average time (*AT*) = 365 × *ED* days. Based on the US EPA guidelines [30], the ILCR was categorized into three risk levels: Negligible (ILCR < 1 × 10^−6^), Cautionary (1 × 10^−6^ ≤ ILCR ≤ 1 × 10^−4^), and Unacceptable (ILCR > 1 × 10^−4^).

The non-carcinogenic risk (NCR) was quantified using the hazard quotient (HQ). The HQ is calculated as the ratio of the estimated exposure level to a reference dose (RfD), which represents a safe exposure threshold below which adverse effects are not expected [31]. It is computed as follows (4) and (5):

(4) $${HQ}_{i}=\frac{C_{i}\times IR}{{RfD}_{i}\times BW}$$

(5) $$HI=\sum_{i=1}^{16}HQ$$

where C_i_ is the concentration of PAH congener i, and RfD_i_ is its corresponding reference dose (mg·kg^−1^). The hazard index (HI), representing the cumulative non-carcinogenic risk from all PAHs, is the sum of the individual HQ values. An HQ or HI value exceeding 1 suggests a potential risk of adverse health effects.

A probabilistic risk assessment was conducted using Monte Carlo simulation. The log-transformed BaP*_eq_* concentrations of the organisms were first tested for normality using the Shapiro–Wilk test, which confirmed that the data approximately followed a log-normal distribution. To determine the appropriate number of iterations, simulations were run with 4000, 7000, 10,000, and 13,000 repetitions. The results stabilized at 10,000 iterations, indicating this was sufficient for achieving convergence. The simulation incorporated both fitted input parameters (*BW*, *IR*, *EF*, *ED*) and fixed parameters (*AT*, *CSF*, *RfD*_i_). The probability distributions fitted to these input parameters are summarized in Table 2 and Table 3.

### 2.5. Data Analysis

Statistical analyses were conducted using SPSS Statistics (v23.0, IBM, Armonk, New York, NY, USA). A one-way ANOVA followed by Tukey’s HSD post hoc test was employed to examine differences in mean PAH concentrations across locations and species, with a significance level of *p* < 0.05. The sources of PAHs were identified using the Positive Matrix Factorization (PMF) model. All graphics were generated with Origin 2022b (OriginLab Inc., Northampton, MA, USA), and the study area map was created using ArcGIS (v10.2, ESRI, Redlands, CA, USA). The probabilistic risk assessment was performed via a Monte Carlo simulation with 10,000 iterations using Oracle Crystal Ball (v11.1.24, USA).

## 3. Results and Discussion

### 3.1. The Bioaccumulation of PAHs in Dominant Aquatic Products

Sixteen PAHs were all detected and quantified in the analyzed samples (Table 4 and Table 5), indicating their widespread occurrence in the aquatic ecosystem of Caohai Lake. According to the contamination classification proposed by Baumard et al., PAH levels in aquatic organisms can be categorized as low (0–200 μg/kg) to moderate (200–500 μg/kg) pollution. In this study, the total concentration of the 16 PAHs in the dominant aquatic products averaged 26.7 μg/kg (wet weight), with a maximum value of 73.3 μg/kg, suggesting only a low level of PAH contamination. The individual PAH compounds were found in the following descending order of concentration: Nap > Phe > Fla > BbF > Chr > Pyr > Acy > Ant > InP > DahA > BaA > BkF > BghiP > BaP > Ace > Flu. Notably, Nap was present at significantly higher levels than the other PAHs, indicating it is the most readily accumulated congener among aquatic species of Caohai Lake. Driven by the pronounced accumulation of Nap, two-ring PAHs were the most dominant homologues, followed by three-, four-, five-, and six-ring PAHs. Overall, the bioaccumulation level exhibited a decreasing trend with increasing ring number, reflecting the stronger bioaccumulation potential of low-molecular-weight PAHs.

The seven carcinogenic PAHs (Σ7CPAHs) had a mean concentration of 5.52 μg/kg, accounting for 20.68% of the total PAHs. A comparative analysis with other regions (Table 4) revealed that the total PAH concentrations among aquatic products of Caohai Lake were significantly lower than those reported in the Yellow River Basin, China, and were generally comparable to those from the Pearl River and Yangtze River Basins. Internationally, the levels in Caohai Lake were considerably lower than those found in Nigeria, Brazil, Italy, and Canada, and were similar to those reported in Argentina and Bangladesh. In summary, the PAH concentrations in the dominant aquatic products from Caohai Lake are relatively low both nationally and internationally. As PAHs are ubiquitous by-products of energy consumption, their level of pollution generally correlates with industrial intensity. Consequently, the relatively low concentrations of PAHs found in fish from Caohai Lake highlight the region’s limited industrial activity and its relatively high ecological quality.

### 3.2. PAH Accumulation in Different Aquatic Species

**Figure 2:**
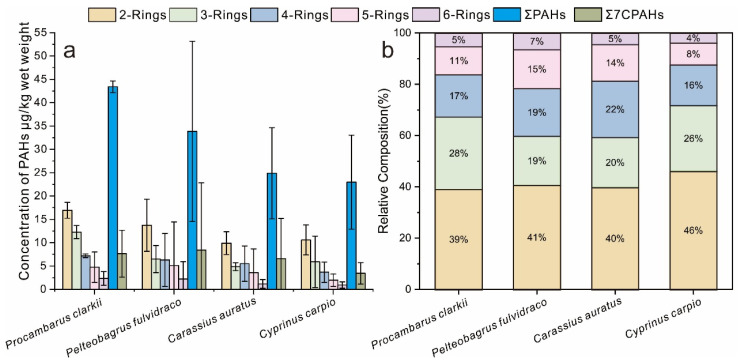
PAH accumulation profiles in different aquatic species: concentrations (**a**) and ratio (**b**) of PAHs by ring number. Note: Only Chr, BaA, BbF, BkF, BaP, DahA, and InP were considered to be carcinogenic PAHs.

Substantial interspecific differences in PAH accumulation patterns were observed among the aquatic species (Figure 2). Regarding total PAH concentrations, the levels decreased in the order crayfish (*Procambarus clarkii*) > yellow catfish (*Pelteobagrus fulvidraco*) > crucian carp (*Carassius auratus*) > common carp (*Cyprinus carpio*). Crayfish accumulated the highest total PAH burden and can thus be considered a high-PAH-accumulating species. However, its accumulation of carcinogenic PAHs was relatively limited and significantly lower than that in yellow catfish. The markedly higher burden of carcinogenic PAHs in yellow catfish identifies it as the species with the greatest potential for accumulating carcinogenic congeners. Since carcinogenic PAHs are predominantly high-molecular-weight compounds, the elevated concentration in yellow catfish is likely attributable to its enhanced accumulation of high-ring PAHs. This is clearly demonstrated by the significantly higher proportions of five- and six-ring PAHs in yellow catfish compared to other species. The greater accumulation of these high-ring PAHs compensates for its lower total PAH accumulation relative to crayfish, resulting in an overall higher carcinogenic potential. Therefore, assessing the carcinogenic risk from fish consumption should not rely solely on total PAH concentrations, but must also consider the composition of the accumulated PAHs, particularly the levels of high-molecular-weight compounds. In contrast, both common carp and crucian carp showed relatively low concentrations of both total and carcinogenic PAHs, indicating a lower capacity for PAH accumulation and classifying them as low-accumulating species. Regarding PAH composition profiles, all species followed a general trend of decreasing accumulation with increasing ring number (and molecular weight). Two-ring PAHs consistently accounted for over 40% of the total PAHs, representing the dominant fraction, whereas six-ring PAHs generally constituted only about 5%, making them the least accumulated group.

### 3.3. The Source Identification of PAHs in Aquatic Species of Caohai Lake

**Figure 3:**
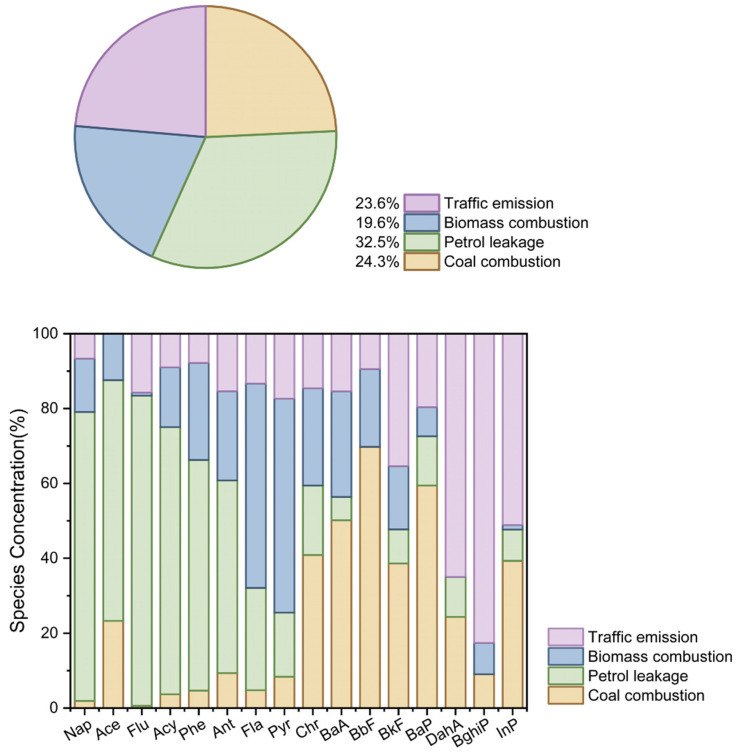
The sources of PAHs among dominant aquatic species in Caohai Lake.

Positive Matrix Factorization (PMF) was applied to identify the sources of PAHs in this study (Figure 3). After testing solutions with two to six factors, a four-factor model was selected as the optimal and most physically interpretable solution, using a random start and 20 iterations. The model exhibited strong agreement between observed and predicted concentrations, with r^2^ values exceeding 0.99 for all PAHs, and all standardized residuals fell within the range of −3 to +3. Factor 1 was characterized by high loadings of high-molecular-weight PAHs, including BaA (50.2%), BbF (69.8%), BaP (59.4%), InP (39.3%), Chr (40.8%), and BkF (38.7%). These compounds are recognized molecular markers for high-temperature combustion processes, particularly coal combustion [13]. Guizhou Province is rich in coal resources, which are extensively used for industrial production and residential heating in forms such as coal-fired power plants, industrial boilers, and household stoves [42]. Although no large-scale industries are located immediately around Caohai Lake, domestic coal usage is widespread in the surrounding communities. The often incomplete combustion of coal in residential settings leads to significant emissions of high-molecular-weight PAHs, such as BaP and BbF. These coal-derived PAHs, many of which are carcinogenic, can enter the aquatic environment via atmospheric deposition or surface runoff, and subsequently accumulate in aquatic organisms [33]. The compositional pattern of Factor 1 aligns well with the regional coal combustion profile; therefore, Factor 1 is identified as a coal combustion source.

Factor 2 exhibited significant contributions to low-molecular-weight PAHs, including Nap (77.2%), Ace (64.3%), Flu (82.8%), Acy (71.4%), Phe (61.6%), and Ant (51.4%). These compounds are primarily volatile and readily degradable, typically originating from direct petroleum release or low-temperature combustion processes [6]. Although Caohai Lake is designated as a wetland nature reserve, substantial agricultural land remains under cultivation in its immediate vicinity [19]. Agricultural activities in the area involve the use of machinery, which potentially leads to fuel evaporation and lubricant leakage, as well as the application of petroleum-based pesticides [15,21]. These practices are likely sources of petroleum-derived PAHs in the watershed. In addition to agricultural inputs, the accumulation of low-molecular-weight PAHs is facilitated by the generally poor hydrodynamic conditions of Caohai Lake [20]. The limited water circulation reduces the volatilization and degradation of these compounds, allowing them to persist and disperse throughout the lake system. This environmental persistence enhances their bioavailability, explaining the notably high levels of low-molecular-weight PAHs, particularly Nap, observed in aquatic organisms. Therefore, Factor 2 is identified as a petroleum contamination source.

Factor 3 was dominated by high loadings of fluoranthene (Fla, 54.6%) and pyrene (Pyr, 57.1%), with moderate contributions to chrysene (Chr, 26.0%), benzo[a]anthracene (BaA, 28.2%), and benzo[b]fluoranthene (BbF, 20.8%). Compared with coal combustion, this factor showed lower contributions to high-molecular-weight PAHs. Previous studies have identified Fla and Pyr as typical tracers of biomass burning, which is often associated with agricultural activities such as the combustion of wild grass, crop residues, and wood [17,43]. As noted earlier, the watershed of Caohai Lake contains extensive farmland both in the immediate vicinity and in the surrounding areas, where the burning of biomass, including weeds, straw, and wood, is common, particularly after harvest seasons or during heating periods. Given the strong agricultural presence in the region, PAHs emitted from biomass burning can be transported through the atmosphere and deposited into the water body [44], thereby enhancing the accumulation of related PAHs in aquatic organisms. Therefore, Factor 3 is attributed to biomass burning sources.

Factor 4 showed prominent contributions to dibenzo[a,h]anthracene (DahA, 65.0%), benzo[ghi]perylene (BghiP, 82.6%), indeno [1,2,3-cd]pyrene (InP, 51.1%), and benzo[k]fluoranthene (BkF, 35.4%). Among these, BghiP and InP are recognized as specific tracers for vehicle emissions, particularly from diesel engines. These high-molecular-weight PAHs are chemically stable and exhibit strong environmental persistence [25]. Although Caohai Lake serves as a key cultural and tourism destination in Weining County, commercial boating has been officially prohibited to protect the ecological environment of this lake. Consequently, the high-molecular-weight PAHs in the watershed of Caohai Lake are likely derived primarily from surrounding road traffic. In reality, the ban on commercial boating has not diminished public enthusiasm for visiting Caohai Lake for recreation. Well-maintained roads around the lake facilitate a substantial influx of private vehicles on weekends, frequently causing severe traffic congestion. Since both diesel and gasoline vehicle emissions release high-molecular-weight PAHs such as BghiP and InP [13,25], the periodic surge in private vehicles inevitably leads to the accumulation of these compounds. This, in turn, enhances their bioaccumulation potential in aquatic organisms. Thus, Factor 4 is identified as a traffic emission source.

Overall, the sources of PAHs accumulated in the dominant aquatic products of the watershed of Caohai Lake, in descending order of contribution, are petroleum leakage > coal combustion > traffic emissions > biomass burning. Petroleum-derived PAHs represent the most significant source. Given that petroleum leakage in the Caohai area is largely associated with agricultural activities in the remaining farmland, strengthening the regulation of agricultural practices around Caohai Lake is of considerable importance for mitigating PAH accumulation in aquatic organisms.

### 3.4. The Health Risk of PAH Bioaccumulation in Aquatic Species

**Figure 4:**
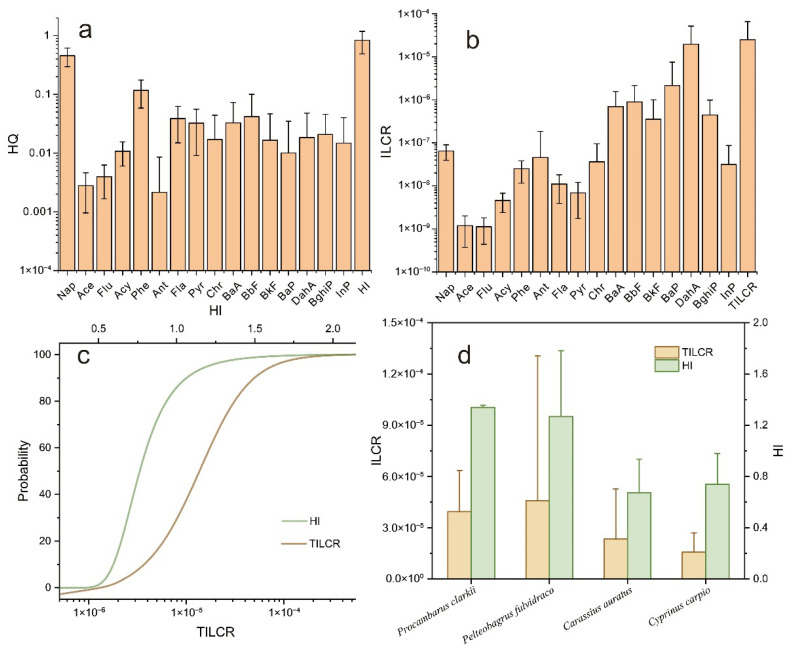
Health risks of PAHs in dominant aquatic products from Caohai Lake. (**a**) Non-carcinogenic risk expressed as the hazard quotient (HQ); (**b**) carcinogenic risk expressed as the incremental lifetime cancer risk (ILCR); (**c**) cumulative probability of total ILCR (TILCR) derived from Monte Carlo simulation; (**d**) ILCR values across different aquatic species.

To assess the potential public health threats posed by PAHs accumulated in dominant aquatic products from the watershed of Caohai Lake, this study calculated both the non-carcinogenic and carcinogenic risks, with the results presented in Figure 4. For non-carcinogenic risk (Figure 4a), the hazard quotient (HQ) values decreased in the order Nap > Phe > BbF > Fla > BaA > Pyr > BghiP > DahA > Chr > BkF > InP > Acy > BaP > Flu > Ace > Ant. Nap showed the highest HQ, indicating it poses the greatest non-carcinogenic risk. Although the average HQ for individual PAHs and the Hazard Index (HI) were both well below 1, the maximum HI value exceeded this threshold. Monte Carlo simulation further revealed that approximately 10% of the aquatic product samples had an HI greater than 1 (Figure 4c), suggesting that non-carcinogenic risk from PAH exposure remains a non-negligible concern. Across species, the HI values decreased in the order crayfish (*Procambarus clarkii*) > yellow catfish (*Pelteobagrus fulvidraco*) > common carp (*Cyprinus carpio*) > crucian carp (*Carassius auratus*). The significantly higher HI values in crayfish and yellow catfish indicate that consumption of these two species is more likely to contribute to non-carcinogenic risk.

For carcinogenic risk (Figure 4b), the incremental lifetime cancer risk (ILCR) values followed the order DahA > BaP > BbF > BaA > BghiP > BkF > Nap > Ant > Chr > InP > Phe > Fla > Pyr > Acy > Ace > Flu. The markedly higher ILCR for DahA identifies it as the PAH with the greatest carcinogenic risk. Among all PAHs, only DahA, BaP, BaA, and BbF had ILCR values exceeding 1 × 10^−6^, yet all remained below 1 × 10^−4^, indicating a detectable but generally acceptable level of carcinogenic risk. The ILCRs of the remaining PAHs were all below 1 × 10^−6^, suggesting negligible carcinogenic risk. Since the ILCRs of DahA, BaP, BaA, and BbF generally surpassed 1 × 10^−6^, the total incremental lifetime cancer risk (TILCR) was also elevated, with a mean value of 2.5 × 10^−5^ (approaching the 1 × 10^−4^ threshold). Monte Carlo simulation confirmed that the TILCR for all samples exceeded 1 × 10^−6^, indicating a measurable carcinogenic risk associated with the consumption of aquatic products from Caohai Lake, though the overall risk remains within acceptable limits. By species, TILCR values decreased in the order yellow catfish > crayfish > crucian carp > common carp. Yellow catfish showed the highest TILCR, implying that its consumption carries a relatively higher carcinogenic risk. Given that both yellow catfish and crayfish exhibited significantly higher HI and TILCR values than crucian carp and common carp, limiting the intake of these two species could help mitigate health risks from excessive PAH exposure.

**Figure 5:**
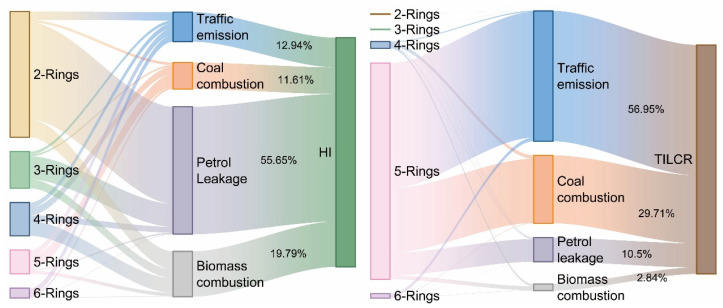
The contribution rates of PAH components and sources to their carcinogenic and non-carcinogenic risks.

Building upon the preceding analysis, the accumulation of PAHs in dominant aquatic products from Caohai Lake does pose measurable health risks. It is therefore essential to clarify the interrelationships between PAH composition, sources, and health risks, as illustrated in Figure 5. For non-carcinogenic risk, the contribution of PAHs to the Hazard Index (HI) decreased in the order two-ring > three-ring > four-ring > five-ring > six-ring PAHs. Notably, two-ring PAHs contributed most significantly to the HI, substantially exceeding the contributions of other homologues. This order aligns perfectly with the overall accumulation pattern of PAHs in the aquatic species, where two-ring PAHs were also the most abundant. This correspondence indicates that the non-carcinogenic risk in Caohai Lake is primarily determined by the total burden of accumulated PAHs, rather than by the presence of specific congeners. In terms of emission sources, contributions to the HI decreased as petroleum leakage > biomass burning > traffic emissions > coal combustion. Petroleum leakage alone accounted for over 50% of the total HI, establishing it as the dominant source of non-carcinogenic risk. The elevated risk from petroleum sources stems mainly from the substantial release of two-ring PAHs into the environment. The abundance of these two-ring PAHs amplifies the impact of petroleum leakage on the HI, while diminishing the relative influence of other sources. Since two-ring PAHs are generally volatile and readily degradable, mitigating non-carcinogenic risk could involve restricting the use of petroleum products around Caohai Lake and enhancing water aeration to promote the degradation of these low-molecular-weight compounds.

In contrast, the contribution to the total incremental lifetime cancer risk (TILCR) decreased in the order five-ring > four-ring > six-ring > three-ring > two-ring PAHs. Strikingly, five-ring PAHs alone contributed more to the TILCR than all other ring classes combined, underscoring their dominant role in carcinogenic risk. This contribution pattern does not mirror the overall accumulation profile of PAHs, revealing that carcinogenic risk is not governed by the total PAH burden, but rather by the accumulation of specific high-risk congeners. Source apportionment for TILCR showed the following decreasing order of contribution: traffic emissions > coal combustion > petroleum leakage > biomass burning. Traffic emissions were responsible for 57% of the TILCR, identifying them as the most significant source of carcinogenic risk. High-molecular-weight PAHs, particularly five-ring compounds that are characteristic of traffic emissions, exhibit strong carcinogenic potency. Given their environmental persistence and low mobility, controlling these high-molecular-weight PAHs requires a focus on source reduction [6].

In summary, while the majority of HQ and HI values for the aquatic products of Caohai Lake were below 1, with only less than 10% of samples indicating potential non-carcinogenic risk, the carcinogenic risk is more widespread. The TILCR exceeded 1 × 10^−6^ in nearly all samples, confirming a measurable carcinogenic risk. Although the average risk remains within acceptable limits (TILCR < 1 × 10^−4^), proactive management is warranted to safeguard ecological and human health. Since traffic emissions are the primary contributor to carcinogenic risk and originate largely from vehicles on the roads encircling the lake, implementing stormwater diversion systems along these roads is strongly recommended. Such measures would reduce the input of traffic-derived PAHs into the lake, thereby mitigating the associated carcinogenic risk accumulated in aquatic products.

It is important to note that the findings of this study are subject to limitations that future research should address. The seasonal dynamics of PAH contamination, driven by variations in temperature and hydrology, can significantly modify both the external pollutant load [45,46] and the internal bioaccumulation processes in aquatic organisms [47], thereby affecting source profiles and health risks. A seasonal monitoring program is therefore needed to capture these critical fluctuations. Furthermore, while this study focused on key edible species, the assessment must be expanded to include a wider range of biota to achieve a truly ecosystem-wide risk evaluation.

## 4. Conclusions

The study revealed a relatively low level of PAH contamination (mean: 26.7 μg/kg) in aquatic products from the Caohai watershed, with a decreasing bioaccumulation tendency as the ring number increased. *Procambarus clarkii* exhibited the highest total PAH concentration, whereas Crayfish accumulated the most carcinogenic PAHs. Source apportionment identified four primary sources—petroleum leakage, coal combustion, traffic emissions, and biomass burning—with petroleum-derived PAHs being the most significant contributor. Although the average HI values were below 1, approximately 10% of the samples posed a potential non-carcinogenic risk, particularly from yellow catfish, and crayfish. The ILCR values of DahA, BaP, BaA, and BbF all exceeded 10^−6^ but remained below 10^−4^, indicating acceptable but non-negligible carcinogenic risks. Notably, the mean TILCR approached 10^−4^, with yellow catfish presenting the highest carcinogenic risk. Petroleum leakage was the dominant contributor to non-carcinogenic risk (>55%), while traffic emissions contributed most to carcinogenic risk (>57%). To mitigate these risks, we recommend implementing stormwater diversion systems along the circular lakeside roads to reduce the input of traffic-derived PAHs.

## Figures and Tables

**Table 1:** Sample information of aquatic products in the Lake Caohai.

Species	Length (cm)	Weight (g)	Feeding	Living	Num
*Pelteobagrus fulvidraco*	13~22.8	25.8~104.7	Omnivorous	Demersal	19
*Carassius auratus*	17.5~21.8	70.5~168.2	Omnivorous	Demersal	23
*Cyprinus carpio*	17~26	68.5~2080	Omnivorous	Demersal	24
*Procambarus clarkii*	10–15	35–50	Omnivorous	Demersal	18

Note: Morphological measurements (body length and weight) were not recorded for Procambarus clarkii due to its limited size variation among individuals.

**Table 2:** The parameters used in health risk assessment of PAHs.

Parameters	Unit	Adult
Average body weight (BW)	kg	60
The concentration of PAH i (C_i_)	mg/kg	-
Exposure duration (ED)	years	(30, 70)
Average time of exposure (AT)	day	365 × ED (Non-carcinogenic)
		365 × 70 (Carcinogenic)
Exposure frequency (EF)	day·year^−1^	365
Carcinogenic slope factor (CSF)	dimensionless	7.3 (Non-carcinogenic)
Intake rate (IR)	g·person^−1^·day^−1^	49.3

Note: The relevant parameters could be found in previous studies [6,10,27,28].

**Table 3:** The toxic equivalence factor, daily intake reference dose and carcinogenic slope factor of PAHs.

PAHs	Abbreviation	*TEF* ^a^	*RfD* ^b^	*CSF* ^c^
Naphthalene	Nap	0.001	0.02	–
Acenaphthene	Acy	0.001	0.06	–
Fluorene	Ace	0.001	0.06	–
Acenaphthylene	Flu	0.001	0.04	–
Phenanthrene	Phe	0.001	0.03	–
Anthracene	Ant	0.01	0.3	–
Fluoranthene	Fla	0.001	0.04	–
Pyrene	Pyr	0.001	0.03	–
Benzo[*a*]anthracene	BaA	0.1	0.03	–
Chrysene	Chry	0.01	0.03	–
Benzo[*b*]fluoranthene	BbF	0.1	0.03	–
Benzo[*k*]fluoranthene	BkF	0.1	0.03	–
Benzo[*a*]pyrene	BaP	1	0.03	7.3
Dibenzo[*a*,*h*]anthracene	DahA	5	0.03	–
Indeno [123-*cd*]pyrene	InP	0.01	0.03	–
Benzo[*ghi*]perylene	BghiP	0.1	0.03	–

Note: ^a^ indicates toxic equivalence factor; ^b^ represents the daily intake reference dose (mg·kg^−1^); ^c^ indicates the carcinogenic slope factor. The relevant parameters could be found in previous studies [6,26,31,32].

**Table 4:** The composition of PAHs in aquatic products of Caohai Lake (unit: μg/kg in wet weight).

Rings	Abb	Range	Mean	SD
2	Nap	6.1~21.4	11.1	3.9
2	Ace	0.1~0.8	0.2	0.1
3	Flu	0.04~0.45	0.19	0.11
3	Acy	0.4~1.8	0.8	0.3
3	Phe	1.3~10.2	4.3	2.1
3	Ant	0.2~14.5	0.8	2.3
4	Fla	0.5~4.7	1.9	1.2
4	Pyr	0.3~4.4	1.2	0.9
4	BaA	0.2~5.9	0.6	1.0
4	Chr	0.2~6.7	1.2	1.5
5	BbF	0.2~10.2	1.5	2.1
5	BkF	0.1~6.5	0.6	1.1
5	BaP	0.03~5.3	0.37	0.91
5	DahA	0.03~6.35	0.67	1.08
6	InP	0.06~5.33	0.8	0.9
6	BghiP	0.05~5.29	0.54	0.92
	ΣPAHs	10.3~73.3	26.7	12.9
	Σ7CPAHs	1.3~41.0	5.5	7.9

Note: Only Chr, BaA, BbF, BkF, BaP, DahA, and InP were considered to be carcinogenic PAHs.

**Table 5:** The content of PAHs in aquatic products of Caohai Lake and that in other regions (unit: μg/kg in wet weight).

Country	Study Area	Year	Matrix	ΣPAHs	Reference
China	Yangtze River Basin	2021	Fish and shellfish	33.91	[33]
China	Yellow River Estuary	2023	Fish	145.9	[34]
China	Pearl River Basin	2024	Fish	42.25	[35]
Argentina	Bahía Blanca estuary	2015–2016	Fish and shellfish	36	[36]
Bangladesh	Bay of Bengal, Chattogram	2024	Fish and shellfish	0.15~67.69	[37]
Brazil	The coast of Pernambuco	2019	Finfish and shellfish	8.71~418	[38]
Nigeria	Makoko Fish Landing Site, Lagos Lagoon, Lagos State	2020	Fish	18,960~45,430	[39]
Canada	Athabasca and Slave Rivers	2011–2012	Fish	4.3~120	[40]
Italy	Adriatic Sea (Mediterranean).	2011	Fish	209.9~227.2	[41]

## Data Availability

Data will be made available on request.
